# Facteurs prédictifs de la polysensibilisation aux allergènes de contact: étude comparative réalisée à l’Unité de Dermato-Allergologie du CHU Farhat Hached de Sousse, Tunisie

**DOI:** 10.11604/pamj.2024.48.40.35295

**Published:** 2024-06-03

**Authors:** Noura Belhadj, Aicha Brahem, Narjes Belhadj Chabbah, Zeineb Athimni, Maher Maoua, Houda Kalboussi, Olfa El Maalel, Souhail Chatti, Najib Mrizak

**Affiliations:** 1 Département de Médecine du Travail, Faculté de Médecine de Sousse, Université de Sousse, Sousse, Tunisie

**Keywords:** Polysensibilisation, dermatite de contact allergique, tests épicutanés, facteurs de risque, Polysensitization, allergic contact dermatitis, patch tests, risk factors

## Abstract

**Introduction:**

la polysensibilisation est souvent définie comme une sensibilisation à trois allergènes de contact ou plus. Les objectifs de notre étude étaient de déterminer la prévalence de la polysensibilisation aux allergènes des tests épicutanés et d'analyser les facteurs associés à une polysensibilisation aux allergènes de la Batterie Standard Européenne en comparaison avec les cas d'oligosensibilisation.

**Méthodes:**

il s'agit d'une étude descriptive transversale rétrospective qui a porté sur tous les patients ayant bénéficié de tests épicutanés à l'Unité de Dermato-Allergologie du Service de Médecine de Travail et de Pathologie Professionnelle du CHU Farhat Hached de Sousse s'étalant sur une période de 10 ans allant du 1^er^ janvier 2009 au 31 décembre 2018.

**Résultats:**

au total, nous avons colligé 464 cas de dermatite de contact au cours de la période d'étude. L'âge moyen des patients était de 38,93 ± 12,52 ans avec une légère prédominance féminine (soit 52,8% des cas). Parmi l'ensemble des patients ayant consulté durant la période d'étude (832 patients), 133 patients avaient de réactions positives à 3 allergènes ou plus soit 16% des patients. En effet, les associations des allergènes les plus fréquemment notées étaient celles de triplet (chrome, cobalt et nickel) chez 15 patients et de triplet (mercaptobenzothiazole, thiuram mix et N-Isopropyl-N-phenyl-4-phenylenediamine (IPPD)) dans 7 cas. Les facteurs prédictifs de polysensiblisation étaient les antécédents personnels d'ulcère de jambe; la localisation des lésions au niveau de thorax et l'aspect érythémato squameux et dysidrosique des lésions.

**Conclusion:**

devant ce phénomène aussi fréquent de polysensibilisation, une collaboration étroite entre dermatologue et médecin du travail pour prévenir ce type d'allergie multiple en passant par une meilleure prise en charge des facteurs de risque personnels et aussi professionnels.

## Abstract (en)

**Introduction:**

polysensitization is often defined as sensitization to three or more contact allergens. The objectives of our study were to determine the prevalence of polysensitization to allergens patch test and to analyze the factors associated with allergen polysensitization of the European Standard Battery in comparison with cases of oligosensitization.

**Methods:**

this is a retrospective descriptive cross-sectional study that included all patients who had undergone patch tests at the Department of Dermato-Allergology of the Department of Occupational Medicine of University Hospital Center Farhat Hached in Sousse spread over 10 years from January 1, 2009 to December 31, 2018.

**Results:**

in total, we collected 464 cases of contact dermatitis during the study period. The average age of patients was 38.93 ± 12.52 years with a slight female predominance (52.8% of cases). Among all the patients who consulted during the study period (832 patients), 133 patients had positive reactions to 3 or more allergens 16% of patients. Concerning, the associations of the most frequently noted allergens were those of triplet (chromium, cobalt, and nickel) in 15 patients and triplet (mercaptobenzothiazole, thiuram mix, and IPPD) in 7 cases. The predictive factors of polysensitivity were the personal history of leg ulcers; the site of the lesions in the thorax and the erythematous squamous and dyshidrotic appearance of the lesions.

**Conclusion:**

this phenomenon is frequent polysensitization, and close collaboration between dermatologists and occupational physicians to prevent this type of multiple allergies through better management of personal and also professional risk factors.

## Introduction

L'allergie de contact est contractée suite à l'exposition à un sensibilisant de contact, suivie d'une dermatite de contact allergique (DCA) lors d'une nouvelle exposition, et elle est généralement diagnostiquée par une réaction positive au patch test (PT). Il a été démontré que des facteurs héréditaires contribuaient à l'acquisition de l'allergie de contact, impliquant une fonction barrière cutanée, un métabolisme cutané et des mécanismes immunologiques [1,2]. C'est une réaction d'hypersensibilité de type retardée déclenchée par un contact direct avec des molécules de faible poids moléculaire dans l'environnement. Son diagnostic repose sur la pertinence de l'histoire clinique et sur les tests cutanés, en particulier le PT à la Batterie Standard Européenne (BSE).

Utilisée en France depuis 1980, la BSE, définie par l'European and Environnemental Contact Dermatitis Research Group (EECDRG), est régulièrement actualisée en fonction des allergènes émergents. Souvent, une seule réaction positive est observée [3]. Cependant, il n'est pas tout à fait rare d'observer de multiples réactions positives à la série initiale, même à trois allergènes différents ou plus. Ce dernier phénomène, appelé «polysensibilisation» (PS) est considéré comme un marqueur phénotypique de la susceptibilité accrue à la sensibilisation de contact [4].

La PS, en termes d'allergies de contact multiples, est souvent définie comme une sensibilisation à trois allergènes ou plus non liés de la BSE [5]. Bien que défini arbitrairement, ce concept est utilisé à la fois pour étudier les facteurs de risque de développement d'allergies de contact multiples. La PS a fait l'objet d'une analyse approfondie par Carlsen. BC *et al*. et Schnuch. A *et al*. [6,7]. L'acquisition de sensibilités multiples est apparemment liée à une exposition élevée à des allergènes environnementaux, à une exposition professionnelle, ou à des dermatoses inflammatoires préexistantes véhiculant des «signaux de danger» citant par exemple une dermatite de contact ou un ulcère de la jambe [8]. La prévalence rapportée de la PS dans les populations testées dépend largement de la population et de la longueur de la série testée, ce qui a donné des prévalences allant de 5% à 20% (ce dernier était basé sur une série de référence composée de 73 à 80 allergènes) [5,9,10].

Les patients atteints de DCA pour un antigène donné courent un risque accru de développer des réactions d'hypersensibilité de type retardée à d'autres antigènes. En plus des ulcères de jambe et de la dermatite de stase, la présence d'une dermatite des mains et d'un eczéma atopique ont été signalées comme facteurs de risque de PS [4]. C'est dans ce cadre que nous avons mené une étude descriptive rétrospective des dossiers médicaux de dermatite de contact des patients ayant bénéficié d'un test épicutané à l'Unité de Dermato-Allergologie du Service de Médecine du Travail et de Pathologie Professionnelle du C.H.U Farhat Hached de Sousse de 2009 à 2018. Les objectifs de notre étude étaient d'étudier les caractéristiques socioprofessionnelles, médicales et cliniques des patients sensibilisés aux allergènes de la BSE de l'Unité de Dermato-allergologie, de déterminer la prévalence de la polysensibilisation aux allergènes des tests épi-cutanés et d'analyser les facteurs associés à cette PS.

## Méthodes

**Conception et cadre de l'étude:** il s'agit d'une étude épidémiologique transversale descriptive rétrospective qui a porté sur tous les patients ayant bénéficié de tests épi-cutanés à l'Unité de Dermato-Allergologie du Service de Médecine de Travail et de Pathologie Professionnelle du CHU Farhat Hached de Sousse s'étalant sur une période de 10 ans.

**Population à l'étude:** au cours de notre enquête, nous avons colligé tous les dossiers des patients ayant consulté à cette unité durant la période allant du 1^er^ janvier 2009 au 31 décembre 2018 pour exploration de DCA et ayant bénéficié de patch tests aux allergènes de la BSE. Seuls les patients ayant présenté des réactions positives aux patchs tests ont été inclus dans notre étude.

**Collecte des données:** le recueil des données s'est basé sur une fiche synoptique remplie à partir des dossiers médicaux et des registres des tests épi-cutanés et comportant des données relatives aux caractéristiques socioprofessionnelles du patient; les antécédents personnels et familiaux allergiques et non allergiques; les habitudes de vie; les données cliniques; les tests épi-cutanés réalisés et leurs résultats et le nombre de réactions positives chez chaque patient.

**Diagnostic de la DCA:** la confirmation du diagnostic de dermatite de contact repose sur l'interrogatoire minutieux, l'examen clinique et les tests épi-cutanés qui visent à reproduire de façon quasi expérimentale les circonstances qui ont donné lieu à l'apparition de l'eczéma de contact. Ils permettent de mettre en évidence un mécanisme allergique de la dermatite et d'identifier l'allergène responsable. Les cas avec des réactions positives aux patchs tests ont été subdivisés selon le nombre des allergènes positifs: une polysensibilisation a été définie par la réaction positive à 3 allergènes ou plus; une oligosensibilisation (OS) a été définie par la réaction positive à un ou 2 allergènes.

**Gestion des données et analyse statistique:** toutes les données des participants ont été anonymisées et traitées de manière strictement confidentielle. Les analyses statistiques ont été réalisées à l'aide du logiciel SPSS dans sa version 23. Nous avons calculé les fréquences et les pourcentages pour les variables qualitatives, ainsi que les moyennes, les écart-types (déviation standard), les médianes et l'étendue des valeurs extrêmes pour les variables quantitatives. Pour la comparaison des moyennes, nous avons utilisé le test « t » de Student pour la comparaison de deux moyennes de séries indépendantes. La comparaison des fréquences a été faite avec le test de Chi-deux de Pearson. Pour l'étude multi -variée, nous avons utilisé une régression logistique binaire multiple lorsque la variable dépendante est qualitative. Pour tous les tests statistiques, le seuil de signification p a été fixé à 0,05.

## Résultats

**Caractéristiques socio-démographiques et professionnelles de la population étudiée:** au total 464 cas de dermatite de contact ont été colligés au cours de la période d'étude, représentant une fréquence de 55,7% de tous les cas testés avec les allergènes de la BSE durant la même période (832 cas). L'âge moyen de notre population était de 38,93 ± 12,52 ans avec des extrêmes allant de 14 à 88 ans. La distribution des cas de DC révélait une légère prédominance féminine (52,8%). Dans notre population d'étude, 9 patients soit 2,1% avaient déclaré être tabagiques et 7 cas (1,5%) alcooliques. Les secteurs d'activité les plus touchés étaient le secteur de bâtiment et travaux publics dans 14,4% des cas, le secteur de la santé dans 10,6% et le secteur textile dans 9,5% des cas. Une minorité des patients étaient sans activité professionnelle soit 18,8% des cas (Tableau 1). L'ancienneté professionnelle moyenne de nos travailleurs était de 12,96 ± 10,15 ans avec des extrêmes allant de 1 à 50 ans. La notion d'atteinte similaire chez d'autres personnes dans les mêmes conditions de travail a été notée chez 57 patients soit 12,3% des cas. Seulement 26,9% des cas portaient des gants lors de ses activités professionnelles. La majorité des patients soit 63% des cas avaient des activités extraprofessionnelles dont la plus notée était l'entretien de la maison (204 cas soit 44%).

**Tableau 1:** répartition de la population d'étude selon le secteur d'activité

Secteur d´activité	Nombre	Pourcentage (%)
**Industrie textile**	44	9,5
**Secteur agricole**	17	3,7
**Industrie électronique**	17	3,7
**Restauration et hôtellerie**	23	5 ,0
**Administration**	31	6,7
**Industrie du cuir et de la chaussure**	7	1,5
**Industrie du bois**	9	1,9
**Secteur du bâtiment et travaux publiques**	68	14,4
**Secteur de métallurgie et de mécanique**	33	7,1
**Secteur de peinture- tôlerie**	17	3,9
**Secteur de santé**	49	10,6
**Secteur de l´éducation**	26	5,6
**Secteur de la coiffure**	7	1,5
**Industrie de caoutchouc**	10	2,1
**Autres**	19	4,0
**Total**	377	81,2

**Caractéristiques médicales de la population étudiée:** concernant les antécédents allergiques personnels, seulement 180 patients avaient des antécédents allergiques (soit 39,7% des cas). Quatre-vingt-sept patients soit 18,8% des cas avaient des antécédents familiaux allergiques alors que 110 patients soit 23,7% des cas avaient des antécédents personnels cutanés dont la plus notée était la mycose cutanée chez 60 cas (soit 12,9%) suivie par la dermatite de contact non allergique chez 26 cas (soit 5,6%). Le diabète était noté chez 15 patients (soit 3,2%) alors que l'antécédent d'ulcère de jambe était noté seulement chez 3 cas (soit 0,6%).

**Données cliniques de la population étudiée:** les mains étaient le siège de prédilection des lésions de DCA avec 71,1% des cas, suivies par les pieds dans 23,1% des cas et les Avant-bras dans 15,1% des cas. L'analyse sémiologique des lésions révélait souvent des aspects variés. L'aspect érythémato-vésiculeux était le plus fréquemment retrouvé. En effet, il a été noté chez 265 patients soit 57,2% des cas suivi par l'aspect érythémato-squameux, noté chez 165 patients soit 35,6% des cas. La durée moyenne d'évolution des lésions cliniques au moment de la pratique des patch-tests était de 56,42 ± 79,76 mois (soit de 4,7 ± 6,2 ans) avec des extrêmes allant de 1 mois à 180 mois (soit de 1 mois à 10 ans). Une amélioration des lésions à l'occasion de l'arrêt de travail (réaction de retrait positive) était décrite par 42,5% des patients (197 cas). Deux cent-dix patients soit 45,3% des cas déclaraient que leurs lésions cutanées semblaient être aggravées par le port de certains vêtements et accessoires dont les plus rapportés étaient les caoutchoucs et les faux bijoux dans 14,7% des cas. Le Tableau 2 résume la sensibilisation de nos patients présentant une DCA aux différents allergènes de la BSE. Les réactions fortement positives (+) représentaient 57,8% des réactions. Les réactions extrêmement positives (+) étaient observées seulement chez 27 patients (soit 5,8%).

**Tableau 2:** résultats de la lecture des patch-tests aux allergènes de la BSE

Allergènes de la BSE	Nombre	Pourcentage (%)
Sulfate de Nickel	169	36,4
Bichromate de potassium (Chrome)	149	32,1
Chlorure de Cobalt	108	23,3
Thiuram mix	145	9,7
Formaldéhyde	51	11,0
Baume de Pérou	7	1,5
N-Isopropyl-N-phénylénediamine (IPPD)	25	5,4
Paraphénylènediamine (PPD)	10	2,2
Fragrance mix I	34	7,3
Kathon CG (5 Chloro-2-métyl-4- isothiazoline)	26	5,6
Primine	8	1,7
Sulfate de néomycine	9	1,9
Colophane	37	8,0
Mercapto mix	16	3,4
Mercaptobenzothiazole	26	5,6
Lactones Sesquiterpéniques	11	2,4
Résine époxy	14	3,0
Dibromodicyanobutane 0,5%	28	6,0
Benzocaîne	15	3,2
Fragrance mix II	21	4,5
Hydroxyméthyl pentylcyclohexene carboxyaldéhyde 5% (HMPCC)	11	2,4
Cliquinol	1	0 ,2
Lanoline	17	3,7
Paraben mix	16	3,4
Quaternium15	13	2,8
Paratertiaire phénol formaldéhyde résine	7	1,5
Methoxy-n-pentyl benzoquinone	1	0,2
Budenoside		
Pivalate de tixocortol	1	0,2
Textile dye Mix	3	0,6

**Polysensibilisation chez la population étudiée:** parmi l'ensemble des patients consultant à l'Unité de Dermato-allergologie du CHU Farhat Hached de Sousse durant la période d'étude (832 patients), 133 patients avaient de réactions positives à 3 allergènes ou plus soit 16 % des patients. En effet, les associations des allergènes les plus fréquemment notées étaient celles de triplet (chrome, cobalt et nickel) avec 15 patients parmi les 133 cas polysensibilisés et de triplet (mercapto benzothiazole, thiuram mix et IPPD) dans 7 cas. Les autres cosensibilisations ou polysensibilisations étaient représentées au niveau du Tableau 3.

**Tableau 3:** principaux résultats des associations entre les différents allergènes de la BSE

Allergènes associées	Nombre des cas	Pourcentage (%)
**Chrome+ Cobalt + Nickel**	15	11,4
**IPPD+ Thiuram mix + Mercaptobenzothiazole**	6	4,5
**Chrome + Cobalt**	15	11,4
**Nickel + Cobalt**	10	7,5
**Chrome + Colophane**	8	6,0
**Thiuram mix + Mercaptobenzothiazole**	7	5,3
**Formaldéhyde + IPPD**	7	5,3
**Thiuram mix + Mercapto mix**	7	5,3
**Thiuram mix + Formaldéhyde**	5	3,8
**Dibromodicyanobutane + Baume de Pérou**	4	3,2
**Fragrance mix I +IPPD**	3	2,3
**Kathon CG + Dibromodicyanobutane**	3	2,3

**Facteurs associés à la polysensibilisation:** nous avons analysé l'association entre la PS et les variables d'intérêt. Ainsi, les sujets âgés de plus de 35 ans présentaient plus de polysensibilisation que les sujets âgés moins de 35 ans sans différence statistiquement significative (p<0,055). L'âge ne semblait pas influencer la PS des allergènes de la BSE en milieu professionnel. Une prédominance féminine a été notée dans les deux groupes avec 52,4% des femmes dans le groupe d'OS versus 56,4% des femmes dans le groupe de PS sans différence statistiquement significative (p=0,44).

**Plusieurs facteurs étaient associés significativement à la PS:** l'antécédent personnel d'ulcère de jambe (p=0,007), la localisation au niveau de thorax (p=0,002), les aspects érythématosquameux et dyshidrosique (p=0,003 et 0,025 respectivement). L'ancienneté professionnelle dans le groupe des oligosensibilisés était de 12,62 ans versus 14,14 ans dans le groupe des polysensibilisés sans différence statistiquement significative (p= 0,19). La durée moyenne d'évolution des lésions cliniques au moment de la pratique des patch-tests n'était pas significativement associée à la PS (p=0,078). La majorité des allergènes de la BSE auquel nos patients étaient sensibilisés était associée de façon statistiquement significative à la PS (Tableau 3). Après régression logistique binaire, il existait une relation statistiquement significative entre la polysensibilisation et l'aspect érythémato squameux (p ajusté=0,01; OR ajusté= 0,53; IC=95% [0,32-0,86]).

## Discussion

Il s'agit d'une étude épidémiologique descriptive rétrospective qui a porté sur tous les patients ayant bénéficié de tests épi cutanés à l'Unité de Dermato-Allergologie du Service de Médecine de Travail du CHU Farhat Hached de Sousse s'étalant sur une période de 10 ans.

**Notre étude présente quelques limites:** un biais de sélection concernant la population d'étude. En effet, les patients étaient seulement ceux qui ont été examinés et testés à l'unité de Dermato-allergologie du Service de Médecine du travail ce qui induit une sous-estimation de la fréquence de la PS. Vu la nature rétrospective de notre étude, basée sur les observations des dossiers, de nombreuses données socioprofessionnelles et médicales peuvent manquer. La BSE a changé de composition plusieurs fois dès le début de l'étude et des réactions faussement négatives ou faussement positives des tests épicutanés pourrait avoir lieu induisant alors une perturbation de l'analyse de nombre des réactions positives aux tests. Toutefois, afin d'éviter la mauvaise interprétation des résultats et diminuer les différentes sources d'erreur liées à l'hétérogénéité méthodologique, tous les PT étaient appliqués et interprétés par des médecins experts avec l'utilisation d'un matériel standardisé, et l'application stricte des recommandations de l'ICDRG [11].

La prévalence rapportée de la PS dans les populations testées dépend largement de la population et de la longueur de la série testée, ce qui a donné des prévalences allant de 5% à 20% [5,9,10]. Parmi l'ensemble des patients consultant à l'Unité de Dermato-allergologie du CHU Farhat Hached de Sousse durant la période d'étude (832 patients), 133 patients avaient de réactions positives à 3 allergènes ou plus soit 16 % des patients. La prévalence de la PS dans le groupe le plus largement rapporté, est celle du réseau d'information des départements de dermatologie (IVDK) en Allemagne, s'est avérée stable autour de 10 % [12,13]. Dans une analyse récente élaborée par Dittmar D *et al*. basée sur les données de PT collectées par le système européen de surveillance des allergies de contact, la prévalence de la PS dans différents pays de l'Europe variait de 4,6% en Italie à 12,7% en Autriche et la prévalence standardisée de l'oligosensibilisation s'élevait à 36,3%. Ainsi, la PS et l'OS varient considérablement d'un pays à l'autre. Cela s'explique principalement par la diversité des critères d'éligibilité pour les PT entre les départements. Une autre explication est la longueur de la série des allergènes de base testée [14].

Dans une étude récente réalisée au Danemark, près de 15% des personnes allergiques ayant réagi aux PT présentaient au moins trois allergies de contact [5]. Ainsi, la PS ne peut pas simplement se produire au hasard, mais il peut s'agir d'un phénomène à part entière. Plusieurs facteurs peuvent contribuer à ce phénomène. Dans notre étude, les sujets âgés de plus de 35 ans étaient plus polysensibilisés que les sujets âgés moins de 35 ans sans différence statistiquement significative. L'âge ne semblait pas influencer la PS aux allergènes de la BSE. Une prédominance féminine a été notée dans les deux groupes avec 52,4% des femmes dans le groupe d'OS versus 56,4% des femmes dans le groupe de PS sans différence statistiquement significative (p=0,44). Ainsi Dittmar D *et al*. en explorant les facteurs prédictifs de la PS, ont conclu que le faible pourcentage de la population masculine dans les groupes oligosensibilisés et polysensibilisés (0,7 et 0,59 respectivement) ont indiqué que les femmes avaient un risque significativement accru d'être sensibilisées dans son ensemble, et en particulier d'être polysensibilisées [14]. De même Alder *et al*. via le réseau d'information des départements de dermatologie (IVDK) dédié à la surveillance clinique des allergies de contact en Allemagne, l'Autriche et la Suisse, sur 230316 consultations documentées entre 1992 et 2014, ils avaient trouvé que le genre n'était pas associé à la PS alors qu'une association faible mais significative avec l'âge a été trouvée [15].

Les secteurs d'activité les plus touchés étaient le secteur du bâtiment et travaux publics dans 14,4% des cas et le secteur de la santé dans 10,6% et le secteur textile dans 9,5% des cas. Dans l'étude d'El Maalel. O, les secteurs d'activité les plus touchés par les dermites de contact étaient le secteur du BTP (14,8%), du textile (10,8%), suivis par les activités de soins et le secteur des services publics [16]. Nous n'avons pas trouvé de différence statistiquement significative en comparant les deux groupes de sensibilisés selon le secteur d'activité (p=0,54). Les antécédents de dermatite de contact constituent un facteur de risque de développer une DCA [17]. L'atopie est une prédisposition personnelle et/ou familiale se manifestant le plus souvent durant l'enfance ou l'adolescence à devenir sensibilisé et à produire des anticorps type IgE spécifiques en réponse à une exposition naturelle à des allergènes, en général des protéines alors que la DCA est une réaction retardée de type IV selon la classification de Gell et Coombs [18,19]. Plusieurs études ont abordé la relation entre l'atopie et la prédisposition aux DCA, et sont contradictoires. Dans notre étude, seulement 180 patients avaient des antécédents d'atopie (soit 39,7% des cas).

Au terme de l'analyse univariée, il n'y avait pas de relation statistiquement significative entre les antécédents d'eczéma atopique et la PS (p=0,42). Dans la littérature, la PS dans les maladies atopiques est associée à une gravité accrue des maladies allergiques [20,21] avec un profil immunologique différent de celui trouvé chez les monosensibilisés et une coïncidence familiale suggérant une base génétique [22,23]. En effet, plusieurs études, citant celle de Carlsen BC *et al*. ont trouvé une association statistiquement significative entre la présence d'une dermatite atopique actuelle ou passée et la PS aux allergènes de la BSE [4]. Dans une récente étude allemande, 38,4% de tous les individus polysensibilisés et 37,8% des individus oligosensibilisés présentaient un eczéma atopique [24]. Il y a quelques décennies, les patients atteints d'eczéma atopique constituaient une partie mineure des populations testées, alors que l'eczéma atopique est aujourd'hui plus courant dans les populations testées [25]. Cette augmentation au cours des années ne peut pas s'expliquer par la génétique. Les déclencheurs présumés de cette augmentation se sont concentrés sur des facteurs environnementaux [26,27]. On peut affirmer que les personnes atteintes d'eczéma atopique ont une réactivité cutanée plus élevée [28-30] et donc développent plus facilement des réactions irritantes et douteuses. Ainsi ces réactions irritantes risquent d'être interprétées faussement en tant que réactions positives, ce qui augmenterait le taux de PS.

Concernant les antécédents non allergiques, seul l'antécédent personnel d'ulcère de jambe était significativement associé à la PS (p=0, 007). Notre étude était en désaccord avec celle de Carlsen. BC qui avait suggéré que les ulcères de jambe ne constituaient pas un facteur de risque de PS [4]. Tavadia S a rapporté que les patients atteints d'ulcère de jambe veineux présentaient des allergies multiples dans 51% des cas et que les groupes d'allergènes les plus fréquents étaient les parfums (30,5%), les antimicrobiens (19,5%), les excipients topiques (19,5%), les accélérateurs de caoutchouc (13,5%) et les corticostéroïdes topiques (8%) [31]. Dans notre série, seule la localisation au niveau de thorax était associée de façon statistiquement significative à la PS (p=0,002). Un site de dermatite était largement admis comme facteur de risque de PS est la partie inférieure de la jambe en raison de l'exposition accrue aux médicaments topiques chez les patients atteints de dermatite de stase des jambes et / ou d'ulcères de jambe chroniques [8]. Dittmar D a conclu que la dermatite des mains est un facteur de risque de PS, ce qui a de nouveau confirmé les précédentes analyses [4,12,15].

Une déclaration de la DCA en tant que maladie professionnelle a été faite pour 112 cas avec 32 cas des polysensiblisés versus 80 cas des oligosensibilisés sans différence statistiquement significative (p=0,72). Dans la littérature, la dermatite professionnelle est un facteur de risque connu de PS; cela est probablement dû à une exposition accrue à de multiples allergènes sur les lieux du travail [32,33]. Ainsi Dittmar. D *et al*. en comparant la PS dans certains pays de l'Europe, ont montré que l'Allemagne, la Finlande et la Pologne ont fourni une proportion de dermatite de contact professionnelle plus élevée que d'autres pays, peut être en raison de fondement des départements spécialisés en dermatologie professionnelle. Un pourcentage plus élevé d'étiologies professionnelles de la dermatite dans la population polysensibilisée que dans la population totale testée peut être observé dans tous ces pays [14]. Les associations des allergènes les plus fréquemment notées étaient celles de triplet (chrome, cobalt et nickel) avec 15 patients parmi les 133 cas polysensibilisées et de triplet mercapto benzothiazole, thiuram mix et IPPD dans 7 cas. Les autres cosensibilisations ou polysensibilisations étaient très diversifiées. Plusieurs travaux [13,34,35] ont étudié les associations entre les allergènes, plus récemment dans une publication de l'IVDK [15]. La plupart des couples peuvent être expliqués soit par une co-sensibilisation suite à une exposition simultanée (par exemple, au moins historiquement, bichromate de potassium et le cobalt dans le ciment), soit par réactivité croisée [p-phénylènediamine (PPD) -N-isopropyl-N'-phényl-4 phénylènediamine (IPPD) et benzocaïne], ou les deux [7,36]. La cosensibilisation peut résulter soit d'une exposition à un produit contenant différents allergènes non liés (par exemple des produits topiques contenant des parfums ou des conservateurs), soit d'une exposition concomitante à différents produits, par exemple dans des professions spécifiques.

Dittmar. D *et al*. ont conclu que les deux paires les plus courantes, à la fois en Europe du Nord / Centrale et en Europe du Sud, sont le mélange de quaternium 15 - formaldéhyde et le mélange carba-mix et thiuram-mix, tous deux avec des OR élevés [14]. Dans une étude précédente, 73% des patients allergiques au formaldéhyde avaient également réagi au quaternium 15 et à l'inverse, 59% des patients positifs pour le quaternium 15 avaient également réagi au formaldéhyde. Des réactions concomitantes entre les dithiocarbamates et le thiuram-mix sont courantes [37]. De même, Dittmar D *et al*. avaient conclu que les ingrédients des médicaments topiques étaient souvent parmi le top 10 des sensibilisants cutanées multiples dans ces deux régions: l'Europe du Nord / Centrale et l'Europe du Sud [14]. Carlsen. BC *et al*. avaient montré qu'une plus grande proportion de patients polysensibilisés par rapport aux patients oligosensibilisés souffre de dermatite chronique [5]. Par conséquent, ce groupe pourrait avoir une exposition plus intense et plus longue à plusieurs médicaments topiques que les patients oligosensibilisés. Dans notre série, l'intensité de la réaction ne variait pas de façon statistiquement significative entre le groupe des poly sensibilisés et celui des oligosensibilisés (p=0,61). Par contre, selon les travaux de Dittmar D *et al*. une corrélation positive a été observée entre le nombre de réactions positives au PT et la proportion de réactions extrêmement positives [14]. De même, l'étude de Brush J a révélé que les sujets présentant des réactions extrêmement positives aux PT étaient susceptibles de réagir davantage à des réactions additionnelles que des personnes présentant des réactions faibles [38]. Une explication possible de ces réactions fortes pourrait être le “syndrome de dos irritable”, bien que l'existence réelle de ce phénomène ait été contestée [39,40]. La meilleure explication des réactions extrêmement positives chez les patients polysensibilisés serait le fait que la PS est un signe de susceptibilité accrue ou également une réactivité accrue. Des exemples peuvent être trouvés dans certaines professions comme les coiffeurs ou dans des sous-groupes de patients exposés à des médicaments topiques. L'exposition à des allergènes souvent trouvés ensemble peut également être à l'origine de la PS comme le cas des parfums (presque toujours des mélanges de nombreux composés simples), des cosmétiques (contenant des parfums et des conservateurs) ou des outils et bijoux (métaux). Inversement, aucun type d'exposition spécifique ne peut être discerné lorsque plusieurs sensibilisations ont été acquises à plusieurs reprises sans relation au fil du temps [41,42].

En résumé, cette étude a montré que les facteurs prédictifs de polysensibilisation étaient les antécédents personnels d'ulcère de jambe, la localisation des lésions au niveau de thorax et l'aspect érythémato-squameux et dyshidrosique des lésions. Ainsi, il semble que les individus polysensibilisés présentent différentes caractéristiques d'une susceptibilité accrue à l'allergie de contact et peuvent représenter un groupe de patients dans lesquels les facteurs de risque génétiques sont plus importants. Néanmoins, la multiplicité des allergènes potentiels dans l'environnement professionnel, justifie l'instauration des mesures de prévention adéquates passant par deux volets: une prévention technique et une prévention médicale.

## Conclusion

La polysensibilisation est souvent définie comme une sensibilisation à trois allergènes de contact ou plus. La prévalence rapportée de la PS dans les populations testées variait de 5% à près de 20 %. L'acquisition de sensibilités multiples est apparemment liée à une exposition élevée à des allergènes environnementaux, à une exposition professionnelle, ou à des dermatoses inflammatoires préexistantes véhiculant des « signaux de danger » citant par exemple une dermatite de contact ou un ulcère de la jambe. Ainsi, devant ce phénomène aussi fréquent de poly sensibilisation, une collaboration étroite entre dermatologues et médecins du travail pour prévenir ce type d'allergie multiple en passant par une meilleure prise en charge des facteurs de risque personnels et aussi professionnels. Des analyses longitudinales devraient porter sur le développement de la PS chez des patients ayant une sensibilisation unique ou car ce phénomène est probablement échelonné.

### Etat des connaissances sur le sujet

- *La polysensibilisation (PS) est souvent définie comme une sensibilisation à trois allergènes ou plus non liés de la Batterie Standard Européenne;*
- *La prévalence rapportée de la PS dans les populations testées dépend largement de la population et de la longueur de la série testée, ce qui a donné des prévalences allant de 5% à 20%;*
- *L'acquisition de sensibilités multiples est apparemment liée à une exposition élevée à des allergènes environnementaux, à une exposition professionnelle, ou à des dermatoses inflammatoires préexistantes véhiculant des « signaux de danger » citant par exemple une dermatite de contact ou un ulcère de la jambe*.

### Contribution de notre étude à la connaissance

- *La prévalence de la PS parmi l'ensemble des patients consultant à l'Unité de Dermato-allergologie du CHU Farhat Hached de Sousse durant la période d'étude était de 16%;*
- *Les facteurs significativement associés à la PS étaient: l'antécédent personnel d'ulcère de jambe, la localisation au niveau de thorax et les aspects érythémato-squameux et dyshidrosique des lésions;*
- *Les associations des allergènes les plus fréquemment notées étaient celles de triplet (chrome, cobalt et nickel) avec 15 patients parmi les 133 cas polysensibilisées et de triplet mercapto-benzothiazole, thiuram mix et IPPD dans 7 cas*.

## References

[1] Menné T, Holm NV (1986). Genetic susceptibility in human allergic contact sensitization. Seminars in Dermatology.

[2] Schnuch A, Westphal G, Mössner R, Uter W, Reich K (2011). Genetic factors in contact allergy--review and future goals. Contact Dermatitis.

[3] Castanedo-Tardan MP, Matiz C, Jacob SE (2011). Contact dermatitis in children - a review of current opinions. Actas Dermo-Sifiliográficas (English Edition).

[4] Carlsen BC, Andersen KE, Menné T, Johansen JD (2009). Characterization of the polysensitized patient: a matched case-control study. Contact Dermatitis.

[5] Carlsen BC, Menné T, Johansen JD (2007). 20 years of standard patch testing in an eczema population with focus on patients with multiple contact allergies. Contact Dermatitis.

[6] Carlsen BC, Andersen KE, Menné T, Johansen JD (2008). Patients with multiple contact allergies: a review. Contact Dermatitis.

[7] Schnuch A, Brasch J, Uter W (2008). Polysensitization and increased susceptibility in contact allergy: a review. Allergy.

[8] Machet L, Couhé C, Perrinaud A, Hoarau C, Lorette G, Vaillant L (2004). A high prevalence of sensitization persists in leg ulcer patients: a retrospective series of 106 patients tested between 2001 and 2002 and a meta-analysis of 1975-2003 data. Br J Dermatol.

[9] Moss C, Friedmann PS, Shuster S, Simpson JM (1985). Susceptibility and amplification of sensitivity in contact dermatitis. Clin Exp Immunol.

[10] Gosnell AL, Schmotzer B, Nedorost ST (2015). Polysensitization and individual susceptibility to allergic contact dermatitis. Dermatitis.

[11] Alikhan A, Cheng LS, Ale I, Andersen KE, Bruze M, Eun HC (2011). Revised minimal baseline series of the international Contact Dermatitis Group ICDRG: Evidence-based approach. Dermatitis.

[12] Schwitulla J, Gefeller O, Schnuch A, Uter W (2013). Risk factors of polysensitization to contact allergens. Br J Dermatol.

[13] Brasch J, Uter W, Geier J, Schnuch A (2001). Associated positive patch test reactions to standard contact allergens. Am J Contact Dermat.

[14] Dittmar D, Uter W, Bauer A, Fortina A, Bircher AJ, Czarnecka-Operacz M (2018). European Surveillance System on Contact Allergies (ESSCA): polysensitization, 2009-20. Contact Dermatitis.

[15] Adler W, Gefeller O, Uter W (2017). Positive reactions to pairs of allergens associated with polysensitization: analysis of IVDK data with machine-learning techniques. Contact Dermatitis.

[16] El Maalel O (2005). Epidémiologie des dermatites de contact professionnelles: à propos d'une étude de la région de Sousse. Mémoire de fin d'étude du mastère de dermatologie professionnelle et de l'environnement. FMS.

[17] Nicholson PJ (2011). Occupational contact dermatitis: known knowns and known unknowns. Clin Dermatol.

[18] Johansson SGO, O 'B Hourihane J, Bousquet J, Bruijnzeel-Koomen C, Dreborg S, Haahtela T (2001). A revised nomenclature for allergy: An EAACI position statement from the EAACI nomenclature task force. Allergy.

[19] Johansson SGO, Bieber T, Dahl R, Friedmann PS, Lanier BQ, Lockey RF (2004). Revised nomenclature for allergy for global use: Report of the Nomenclature Review Committee of the World Allergy Organization, October 2003. J Allergy Clin Immunol.

[20] Kim KW, Kim EA, Kwon BC, Kim ES, Song TW, Sohn MH (2006). Comparison of allergic indices in monosensitized and polysensitized patients with childhood asthma. J Korean Med Sci.

[21] Schäfer T, Heinrich J, Wjst M, Adam H, Ring J, Wichmann H-E (1999). Association between severity of atopic eczema and degree of sensitization to aeroallergens in schoolchildren. J Allergy Clin Immunol.

[22] Pène J, Rivier A, Lagier B, Becker WM, Michel FB, Bousquet J (1994). Differences inIL-4 release by PBMC are related with heterogeneity of atopy. Immunology.

[23] Yoo Y, Yu J, Kim DK, Choi SH, Koh YY (2005). Coincidence of atopy and its profile (monosensitization/polysensitization) between sibling pairs. Ann Allergy Asthma Immunol.

[24] Heine G, Schnuch A, Uter W, Worm M (2006). Type-IV sensitization profile of individuals with atopic eczema: results from the Information Network of Departments of Dermatology (IVDK) and the German Contact Dermatitis Research Group (DKG). Allergy.

[25] Cronin E, Bandmann HJ, Calnan CD, Fregert S, Hjorth N, Magnusson B (1970). Contact dermatitis in the atopic. Acta Derm Venereol.

[26] Diepgen T (2005). Epidemiology and job-related problems for the eczema patient. Acta Derm Venereol.

[27] Diepgen TL (2001). Atopic dermatitis: the role of environmental and social factors, the European experience. J Am Acad Dermatol.

[28] Löffler H, Effendy I (1999). Skin susceptibility of atopic individuals. Contact Dermatitis.

[29] Nassif A, Chan SC, Storrs FJ, Hanifin JM (1994). Abnormal skin irritancy in atopic dermatitis and in atopy without dermatitis. Arch Dermatol.

[30] Tupker RA, Pinnagoda J, Coenraads PJ, Nater JP (1990). Susceptibility to irritants: role of barrier function, skin dryness and history of atopic dermatitis. Br J Dermatol.

[31] Tavadia S, Bianchi J, Dawe RS, Mc Evoy M, Wiggins E, Hamill E (2003). Allergic contact dermatitis in venous leg ulcer patients. Contact Dermatitis.

[32] Cahill J, Williams JDL, Matheson MC, Palmer AM, Burgess JA, Dharmage SC (2017). Occupational Contact Dermatitis: A review of 18 years of data from an occupational dermatology clinic in Australia. Safe Work Australia.

[33] Diepgen TL, Kanerva L (2006). Occupational skin diseases. Eur J Dermatol.

[34] Hegewald J, Uter W, Pfahlberg A, Geier J, Shnuch A (2005). A multifactorial analysis of concurrent patch-test reactions to nickel, cobalt, and chromate. Allergy.

[35] Uter W, Larese Filon F, Rui F, Balato A, Wikinson M, Krecisz B (2016). ESSCA results with nickel, cobalt and chromium, 2009-2012. Contact Dermatitis.

[36] Thomas BR, White IR, Mc Fadden JP, Banerjee P (2014). Positive relation ship-intensity of response to p-phenylene diamine on patch testing and cross-reactions with related allergens. Contact Dermatitis.

[37] De Groot AC, Blok J, Coenraads P (2010). Relationship between formaldehyde and quaternium-15 contact allergy. Influence of strength of patch test reactions. Contact Dermatitis.

[38] Brasch J, Schnuch A, Uter W (2006). Strong allergic patch-test reactions may indicate a general disposition for contact allergy. Allergy.

[39] Cockayne SE, Gawkrodger DJ (2000). Angry back syndrome is often due to marginal irritants: a study of 17 cases seen over 4 years. Contact Dermatitis.

[40] Crépy M-N (2012). Eczéma professionnel, actualités 2012. Revue française d'allergologie.

[41] Mangelsdorf HC, Fleischer AB, Sheretz EF (1996). Patch testing in an aged population without dermatitis: high prevalence of patch test positivity. Am J Contact dermat.

[42] Magnusson B, Möller H (1979). Contact allergy without skin disease. Acta Derm Venereol Suppl (Stockh).

